# National Benchmarks for the Efficacy of Trigger Finger and the Risk Factors Associated With Failure

**DOI:** 10.5435/JAAOSGlobal-D-22-00198

**Published:** 2023-02-03

**Authors:** Jennifer Lewis, Henry Seidel, Lewis Shi, Jennifer Wolf, Jason Strelzow

**Affiliations:** From the Department of Orthopaedic Surgery and Rehabilitation Medicine, University of Chicago Medicine, Chicago, IL (Ms. Lewis); Pritzker School of Medicine at The University of Chicago, Chicago, IL (Mr. Seidel); and Department of Orthopaedic Surgery and Rehabilitation Medicine, The University of Chicago, Chicago, IL (Dr. Shi, Dr. Wolf, and Dr. Strelzow).

## Abstract

The purpose of this study was to compare the efficacy of single and multiple corticosteroid injections used for symptomatic trigger finger. The rates of subsequent injections and the rate of tendon sheath release are reported along with the identification of risk factors correlated with failure of injection.

**Methods:**

A retrospective review of a national healthcare database was conducted identifying patients with a diagnosis of trigger finger or thumb. Inclusion required a tendon sheath injection on the same day or within six weeks of diagnosis. Patient cohorts were further stratified based on treatment success and those requiring additional injections within 6 months or surgery within 1 year of initial diagnosis.

**Results:**

Thirty-one thousand seven hundred fifty-one patients met inclusion criteria and underwent an initial injection within the study period. The efficacy of initial, second, and third injection was 66.3%, 79.4%, and 79.6%, respectively. Of the patients who failed an injection, 9.4% had tendon sheath release after a primary injection, 23.1% had surgery after a second injection, and 30.4% had surgery after a third injection. Only obesity (OR 1.2; *P* < 0.0001) and concomitant diagnosis of carpal tunnel syndrome (OR 1.4; *P* < 0.0001) were found to be significant for injection failure on multivariate logistic regression analysis.

**Discussion:**

Overall corticosteroid injections were effective in greater than 65% of patients. This information may help guide treatment practice because there seems to be continued additional benefit to repeat corticosteroid injections after injection failure.

Stenosing tenosynovitis, commonly known as trigger finger, is a common condition with a 2% to 3% lifetime risk of occurrence.^1^ Trigger finger most often occurs idiopathically with an inconsistency between the diameter of the tendon sheath and the flexor tendon of the affected finger; this leads to a narrowing of the sheath and prevents the tendon from moving smoothly through the sheath.^2-4^ Pain may occur with flexion or extension, and patients often report a clicking or locking sensation with finger movement, stiffness that is worse in the morning, and swelling in the metacarpophalangeal joint.^5,6^ Established risk factors for trigger finger include female sex, age between 40 and 60 years, carpal tunnel syndrome, rheumatoid arthritis, and diabetic comorbidity.^5,7^ Patients used in jobs that require repeated gripping are also at a high risk of developing trigger finger.^5,7,8^

The goals of trigger finger treatment center around restoring functional ability and eliminating the catching sensation on extension and flexion of the finger.^9,10^ Nonprocedural approaches, including nonsteroidal anti-inflammatory agents, splinting of the finger, and massage, are often the first treatment for patients with mild symptoms.^11,12^ In patients with more severe symptoms, or when mild symptoms do not resolve after conservative therapy, corticosteroid injection may be indicated.^13-15^ Patients may require additional injections where injections are typically at least 3 months apart.^16^ Surgical intervention with percutaneous, endoscopic, or open A1 pulley release may be indicated in patients with trigger finger refractory to conservative management and corticosteroid injections.^17-19^ In more than 90% of cases, surgical intervention results in successful resolution of symptoms.^2,20^

Although previous literature has focused on how demographics, comorbidities, and injection technique affect the efficacy of the injection,^21-23^ the literature is less clear regarding patients who have failed corticosteroid injections and comparing their success with different follow-up treatment options.

The purposes of this study were threefold to (1) quantify the rate of success and failure in patients who had one, two, or three subsequent corticosteroid injections, (2) quantify the rate of failure experienced after subsequent injections or conversion to surgical tendon sheath incision after corticosteroid injections, and (3) identify the factors correlated with patients undergoing subsequent injections or tendon sheath incision.

## Methods

### Database

This retrospective database study used the Mariner National Insurance database, which contains 30 million patient records from 2010 to 2019 and is accessible through the PearlDiver Research Program. The data in PearlDiver are Health Insurance Portability and Accountability Act compliant, and thus, this study was determined to be exempt from further Institutional Review Board review.

### Study Group

Patients with a diagnosis of stenosing tenosynovitis were identified in the database using the International Classification of Diseases, 10th Revision (ICD-10 codes) (Appendix S1, http://links.lww.com/JG9/A256). Patients were excluded if they were no longer enrolled in their insurance carrier 1 year after their trigger finger diagnosis and thus were no longer in the database. Patients were included in this study if they received a corticosteroid injection within six weeks of their trigger finger diagnosis. Injections were identified using Current Procedural Terminology codes (Appendix S1, http://links.lww.com/JG9/A256).

Overall rates of single and subsequent injections as well as conversion to surgical management were recorded from the date of the diagnosis. We elected to use a 1-year follow-up time point to limit the likelihood of multiple digit involvement. Success from a corticosteroid injection was defined as the lack of need for additional injections up to 6 months after diagnosis and surgery up to 1 year after diagnosis. The 6-month efficacy of subsequent (second or third) injections was reviewed for patients who underwent additional injections within the initial 6-month period. Patients were excluded if they had subsequent treatments beyond the time frame of interest. Patient demographics and comorbidities were also reviewed to assess for risk factors associated with failure (Appendix S2, http://links.lww.com/JG9/A256).

### Statistical Analysis

Categorical data were analyzed by univariate tests, and the predictability of injection failure (subsequent injection or surgery) of each demographic and comorbidity was examined by multivariate logistic regression analysis. Odds ratios (OR) with 95% confidence intervals were computed for all risk factors. All statistical analysis was done through the R statistical package available through the PearlDiver software. Statistical significance was determined at *P* ≤ 0.05.

## Results

**Figure 1:**
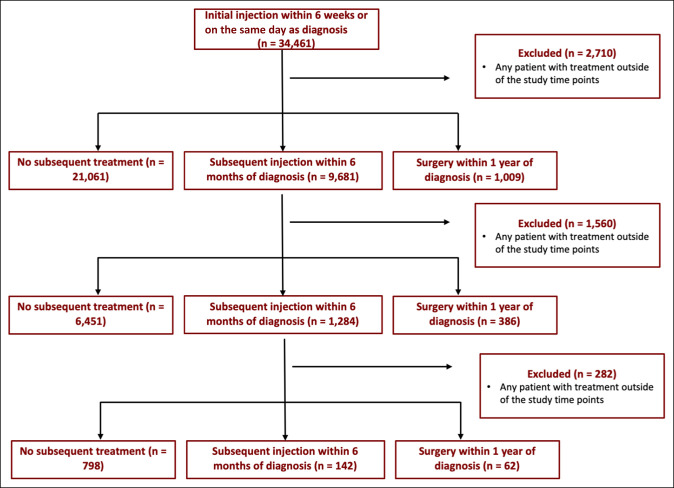
Flow diagram illustrating the outcomes of one, two, and three injections.

**Figure 2:**
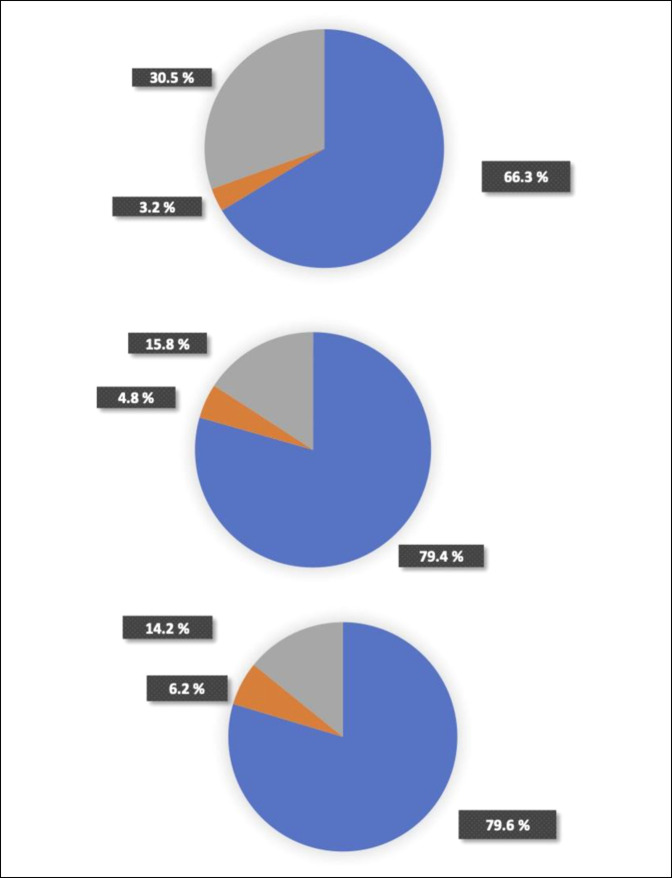
Pie charts illustrating the patients with success (blue), injection (grey), and surgery (orange) among those who received one (top), two (middle), and three (bottom) injections.

Of the 158,705 patients with a diagnosis of trigger finger or trigger thumb in the database, 131,344 had a 1-year follow-up. From this group, a total of 31,751 patients met our inclusion criteria and were identified in the database (Figure 1). Of those patients, 1,009 (3.2%) had surgery within 1 year, 9,681 (30.5%) had a subsequent injection within 6 months, and 21,061 (66.3%) did not undergo subsequent treatment (Figure 2).

Using univariate and multivariate logistic regression analysis, obesity (univariate: OR 1.3, *P* < 0.0001; multivariate: OR 1.2, *P* < 0.0001), tobacco use (univariate: OR 1.2, *P* < 0.0001; multivariate: OR 1.2, *P* < 0.0001), carpal tunnel (univariate: OR 1.5, *P* < 0.0001; multivariate: OR 1.4, *P* < 0.0001), and hypothyroidism (univariate: OR 1.1, *P* = 0.045; multivariate: OR 1.1, *P* = 0.039) were found to be significantly associated with increased risk for failure of initial injection (Table 1). The factors of age older than 65 years (univariate: OR 0.82, *P* < 0.0001; multivariate: OR 0.86, *P* < 0.0001) and ischemic heart disease (univariate: OR 0.94, *P* = 0.024; multivariate: OR 0.94; *P* = 0.037) were significantly associated with decreased risk for failure of first injection. Diabetes was also associated with decreased risk for failure of first injection in the univariate (univariate: OR 1.3, *P* < 0.0001). Female sex, diabetes, and hypertension were not found to be significantly associated with injection outcome in the multivariate logistic regression analysis (Table 1).

**Table 1:** Univariate and Multivariate Analysis Illustrating the Effect of Demographics on First Injection Outcomes

First Injection			Univariate		Multivariate	
Variables	N	% Failed First injection	Odds Ratio	P	Odds Ratio	P
Total	31,751	33.7				
Age > 65	14,171	30.6	0.82 (0.78-0.85)	**<0.0001**	0.86 (0.82-0.90)	**<0.0001**
Sex (female)	19,888	33.1	1.00 (0.95-1.04)	0.92	0.96 (0.92-1.00)	0.064
Diabetes	14,058	33.4	1.07 (1.03-1.12)	**0.0017**	1.04 (0.99-1.09)	0.82
Hypertension	22,779	32.7	0.99 (0.95-1.04)	0.71	0.97 (0.92-1.03)	0.33
Obesity	11,169	35.5	1.3 (1.2-1.3)	**<0.0001**	1.2 (1.1-1.2)	**<0.0001**
Tobacco use	6,168	35.0	1.2 (1.2-1.3)	**<0.0001**	1.2 (1.1-1.2)	**<0.0001**
Ischemic heart disease	6,753	31.3	0.94 (0.90-0.99)	**0.024**	0.94 (0.89-1.00)	**0.037**
Carpal tunnel	6,234	37.8	1.5 (1.4-1.5)	**<0.0001**	1.4 (1.4-1.5)	**<0.0001**
Hypothyroidism	5,909	33.8	1.1 (1.0-1.1)	**0.045**	1.1 (1.0-1.1)	**0.039**

Bold indicates statistical significance for P ≤ 0.05.

Of the 8,121 patients who received a second injection within 6 months, 386 (4.8%) patients had surgery within 1 year of diagnosis, 1,284 (15.8%) obtained an additional injection within 6 months of diagnosis, and 6,451 patients (79.4%) did not endure further treatment (Figure 2).

In univariate and multivariate logistic regression analysis, only carpal tunnel (univariate: OR 1.7, *P* < 0.0001; multivariate: OR 1.7; *P* < 0.0001) was found to be significantly associated with increased risk for failure of second injection. By univariate analysis, obesity (univariate: OR 1.12, *P* = 0.018) and tobacco use (univariate: OR 1.13, *P* = 0.031) were found to be significantly associated with increased risk for failure of second injection and age older than 65 years (univariate: OR 0.88; *P* = 0.011) was found to be associated with decreased risk of second injection failure. In the multivariate, hypertension (multivariate: OR 0.87, *P* = 0.016) was associated with decreased risk of second injection failure (Table 2).

**Table 2:** Univariate and Multivariate Analysis Illustrating the Effect of Demographics on Second Injection Outcomes

Second Injection			Univariate		Multivariate	
Variables	N	% Failed Second Injection	Odds Ratio	P	Odds Ratio	P
Total	8,121	20.6				
Age > 65	3,447	19.03	0.88 (0.80-0.97)	**0.011**	0.92 (0.83-1.02)	0.13
Sex (female)	5,122	19.62	0.96 (0.87-1.06)	0.43	0.92 (0.83-1.02)	0.11
Diabetes	3,698	20.34	1.04 (0.95-1.14)	0.39	1.05 (0.95-1.16)	0.37
Hypertension	5,835	19.79	0.91 (0.82-1.01)	0.064	0.87 (0.77-0.97)	**0.016**
Obesity	3,037	20.84	1.12 (1.02-1.23)	**0.018**	1.08 (0.98-1.20)	0.13
Tobacco use	1,672	21.65	1.13 (1.01-1.26)	**0.031**	1.08 (0.96-1.21)	0.18
Ischemic heart disease	1,667	20.40	1.04 (0.93-1.17)	0.48	1.07 (0.95-1.21)	0.28
Carpal tunnel	1,723	27.05	1.7 (1.6-1.9)	**<0.0001**	1.7 (1.5-1.9)	**<0.0001**
Hypothyroidism	1,555	19.81	1.05 (0.93-1.18)	0.43	1.06 (0.94-1.20)	0.34

Bold indicates statistical significance for P ≤ 0.05.

Of the 1,002 patients who underwent a third injection within 6 months of diagnosis, 62 (6.2%) had surgery within 1 year, 142 (14.2%) received an additional injection within 6 months, and 798 (79.6%) experienced no further treatment (Figure 2).

By univariate and multivariate logistic regression analysis, obesity (univariate: OR 1.6, *P* = 0.0008; multivariate: OR 1.6, *P* = 0.002) and carpal tunnel (univariate: OR 1.7, *P* = 0.0002; multivariate: OR 1.6, *P* = 0.001) were found to be significantly associated with increased risk for third injection failure. Hypertension (univariate: OR 0.75, *P* = 0.043; multivariate: OR 0.66, *P* = 0.009) was associated with a decreased risk of third injection failure (Table 3).

**Table 3:** Univariate and Multivariate Analysis Illustrating the Effect of Demographics on Third Injection Outcomes

Third Injection			Univariate		Multivariate	
Variables	N	% Failed Third Injection	Odds Ratio	P	Odds Ratio	P
Total	1,002	20.4				
Age > 65	423	17.26	0.82 (0.62-1.07)	0.14	0.92 (0.69-1.23)	0.59
Sex (female)	626	20.77	1.16 (0.88-1.53)	0.30	1.02 (0.76-1.36)	0.91
Diabetes	455	19.56	0.98 (0.75-1.28)	0.89	0.96 (0.72-1.29)	0.80
Hypertension	716	18.30	0.75 (0.57-0.99)	**0.043**	0.66 (0.48-0.90)	**0.009**
Obesity	386	23.06	1.6 (1.2-2.0)	**0.0008**	1.6 (1.2-2.1)	**0.002**
Tobacco use	215	20.93	1.08 (0.78-1.46)	0.64	1.04 (0.75-1.43)	0.82
Ischemic heart disease	208	23.08	1.16 (0.84-1.59)	0.36	1.31 (0.92-1.84)	0.13
Carpal tunnel	277	26.35	1.7 (1.3-2.2)	**0.0002**	1.6 (1.2-2.1)	**0.001**
Hypothyroidism	198	22.73	1.28 (0.93-1.75)	0.124	1.25 (0.89-1.73)	0.20

Bold indicates statistical significance for P ≤ 0.05.

Of the patients who failed treatment, 9.4% of patients with one failed injection, 23.1% of patients with two failed injections, and 30.4% of patients with three failed injections elected to undergo surgery instead of continuing management with corticosteroid injections (Table 4).

**Table 4:** Success and Failure Rates Among Patients Receiving One, Two, and Three Injections

Number of Injections	N	% Total Successful	% Total Patients With Subsequent injection	% Total Patients With Surgery	% Failed Patients With Subsequent Injection	% Failed Patients With Surgery
1	31,751	66.3	30.5	3.2	90.6	9.4
2	8,121	79.4	15.8	4.8	76.9	23.1
3	1,002	79.6	14.2	6.2	69.6	30.4

Bold indicates statistical significance for *P* ≤ 0.05.

## Discussion

The efficacy of corticosteroid injections in the management of stenosing tenosynovitis is of interest to hand surgeons and has been assessed by multiple studies. However, previous literature has been limited by smaller sample sizes and single-institution studies.^19,24,25,26,27,28,29,30,31,32^ The range of reported success after corticosteroid injections is broad, making more specific suggestions for patients difficult to interpret. These variations may affect the decision-making and potential outcomes of individual physicians, medical centers, and regional practices. We sought to harness the power of a national insurance claims database to better understand the trends and efficacy of corticosteroid injections on a large scale.

In this study, we report a 66.3% efficacy rate after an initial injection where failure was defined as subsequent injections within 6 months or proceeding to surgery within 1 year. Patients who went on to a second or third injection within the initial 6-month period demonstrated efficacy rates of 79.4% and 79.6% at 6 months postdiagnosis, respectively. Furthermore, we observed an increasing percentage of patients undergoing surgery after each additional injection.

The existing literature has reported a wide range of success after corticosteroid injections; 49% to 84% after one injection,^24,25,27-29^ 23% to 86% after two injections, and 4% to 73.9% after three injections.^15,19,24,25,30^ Our results fit within these previous findings for the first and second injection, whereas the third is slightly above the range. The variation seen before the literature may be the result of variable durations of follow-up, as well as using various definitions of failure after corticosteroid injections, ranging from no improvement of symptoms to receiving a subsequent injection or surgery. A recent study of 292 patients at a single tertiary center by Dardas et al^26^ used a similar definition of failure as our study, defined as either subsequent corticosteroid injection or surgical treatment. They reported 39% (111 of 284) of patients achieved long-term success after two injections and another 39% (24 of 62) of patients resolved their trigger after three injections with a minimum follow-up of 1.5 years. Although we also observed a similar rate of efficacy after both the second and third injections, the rates reported in our study of 79.4% and 79.6% were significantly higher than in the study of Dardas et al despite similar time frames of follow-up.

The relative rates of success increased after additional corticosteroid injections, and the absolute rates of success were high after all three injections. This finding suggests that continuing corticosteroid treatment can be an effective tool for ongoing symptoms and to avoid progression to surgical release. However, due to complications associated with corticosteroid injections, physicians typically do not perform more than two or three injections on a given finger, although variation in practice does occur.^30,33,34^ Complications of corticosteroid injections include the increase of blood glucose levels in diabetic patients, skin or fat atrophy, and spontaneous rupture of fascial and tendons.^34,35,36,37^ Regardless, the more injections patients require for trigger digits, the more likely they are to proceed to surgical management after a failed injection. Our findings, however, suggest that perhaps a continued trial of corticosteroid management may be efficacious in a large portion of patients, preventing the need for surgery at the 1-year time point.

The majority of previous studies that have investigated risk factors for failure of corticosteroid injections in stenosing tenosynovitis have focused on common comorbidities such as diabetes and patient age.^23,26,31^ This study determined that patients with carpal tunnel syndrome were markedly more likely to fail their first, second, or third corticosteroid injections and obesity was a similar albeit smaller risk factor. The literature indicates that trigger finger may occur in combination with carpal tunnel surgery and that these two diagnoses are frequently seen in the same patient.^38,39,40,41^ El-Hadidi et al^42^ offered an explanation for the association between carpal tunnel surgery and trigger finger; he described that the angle between the A1 pulley and the flexor tendon increases during carpal tunnel surgery, and this could precipitate greater friction between these two and potentially lead to the onset of a trigger digit. In addition, patients older than 65 were more likely to have a successful outcome after the first injection. This result may be explained by older patients being less likely to endure subsequent elective procedures compared with younger patients or be willing to ‘live with’ their trigger finger symptoms. Notably, diabetes was not a significant risk factor of injection failure in our analysis. Although some previous studies, such as the one by Chang et al, suggest that diabetes is a risk factor, other studies by Baek et al and Grandizio et al determined that diabetics had equal efficacy of initial corticosteroid injections to nondiabetics.^23,31,43^ Our findings confirm this with a significantly larger population-based analysis.

Overall, most of the comorbidities investigated in this study did not result in decreased efficacy of corticosteroids. Thus, continued corticosteroid treatment may be appropriate in most of the patients. Despite this, the presence of obesity and concomitant or prior carpal tunnel syndrome may be important comorbidities to consider when deciding to pursue continued corticosteroid treatment or electing for surgical management.

## Limitations

The authors recognize several limitations to this study. Due to the claims-based reporting of the PearlDiver database, we did not have a granular clinical understanding of the patients and have limited insight into the multitude of decision-making factors related to undergoing the various possible treatment options. In Pearldiver, laterality could not be determined for the current procedural terminology codes. Hence, we elected to examine a relatively short follow-up period to avoid possible confounding issues of laterality and subsequent additional digit involvement, which cannot be controlled for within the national database. Rates of adjacent trigger finger are generally low within 1 year and have been reported in the literature; Rozental et al^44^ reported rates of 12% within 12.6 months, and thus, we felt a short time frame would limit this potential confounder. Although this methodology still has some margin of error, the short follow-up attempts to ensure future injections and surgical procedures were done on the ipsilateral hand or finger for the vast majority of patients. However, the shorter follow-up may have resulted in the injection efficacy of this study being in the upper range of the literature. Sheikh et al^32^ expressed that the rate of injection failure increased dramatically from 6 months at 22% to 54% at 1 year. Finally, although the authors reported clinical significance for hypothyroidism and ischemic heart disease after a first injection, these OR values were nearing 1.00. These findings suggest statistical significance, which may be due to the large sample size rather than clinical significance for these demographics. In addition, although OR values less than 0.9 and greater than 1.1 seem predictive of outcome, it is possible that OR values within this range are also not clinically significant. Therefore, interpretation must be carefully considered regarding all risk factors whose OR values are close to 1.00. In light of these limitations, this study still clarified the short-term effectiveness of initial and multiple corticosteroid injections in a national population and demonstrated that patients experiencing several failed corticosteroid injections are more likely to undergo surgery with each additional failed injection.

## Conclusion

The rate of initial injection efficacy was 66.3%, whereas the rates of efficacy for second and third injections were 79.4% and 79.6%, respectively, suggesting that patients should consider additional corticosteroid injections after prior failure. Of the patients who failed corticosteroid management, 9.4% with an initial failed injection, 23.1% with two failed injections, and 30.4% with three failed injections decided to undergo tendon sheath incision; thus, patients are more likely to undergo surgery with each additional failed injection. Overall comorbidities do not seem to play a role in injection success rates; however, obesity and carpal tunnel may be associated with lower overall efficacy from corticosteroid treatment.
